# HIV epidemic and needs beyond fast-track cities: a transmission network analysis to study the dynamics of HIV clusters in a French region near Paris

**DOI:** 10.1128/spectrum.02542-25

**Published:** 2026-04-22

**Authors:** Nived Collercandy, Marc-Florent Tassi, Antoine Chaillon, Leslie Grammatico-Guillon, Emeline Laurent, Laurent Hocqueloux, Thierry Prazuck, Clémence Guillaume, Jérôme Guinard, Adrien Lemaignen, Guillaume Gras, Karl Stefic

**Affiliations:** 1 Division of Tropical and Infectious Diseases, University Hospital of Tours, Tours, France; 2 INSERM U1259 MAVIVHe, University of Tours, Tours, France; 3 Public Health and Prevention Division, Epidemiology Unit for Clinical Data in Centre-Val de Loire (EpiDcliC), University Hospital of Tours, Tours, France; 4 Division of Infectious Diseases, University of California San Diego 8784, La Jolla, California, USA; 5 Division of Tropical and Infectious Diseases, University Hospital of Orléans, Orléans, France; 6 Laboratoire Interdisciplinaire pour l'Innovation et la Recherche en Santé d’Orléans (LI²RSO), University of Orléans 27047, Orléans, France; 7 Regional Coordination Committee for Combating HIV (COREVIH) Centre-Loire Valley, Orléans, France; 8 Department of Microbiology-Virology-Parasitology, University Hospital of Orléans, Orléans, France; 9 Department of Virology, HIV National Reference Center, University Hospital of Tours, Tours, France; University of Brescia, Brescia, Italy

**Keywords:** molecular epidemiology, phylogenetic, transmission cluster, HIV

## Abstract

The Centre-Loire Valley region is a low-density demographic and medical region near the fast-track city of Paris, with persistent elevated rates of positive HIV testing. We investigated the role and characteristics of transmission clusters in the HIV dynamics in this region for appropriate local response. HIV pol gene sequences collected in Centre-Loire Valley over a decade (2010–2020) were included in a phylogenetic analysis combined with epidemiological data. Putative transmission clusters were inferred using HIV Transmission Cluster Engine methods. Risk factors of being part of a cluster were studied using multivariate logistic regression models. Of the 1,305 participants, 579 (44%) were born in France, mainly from sub-Saharan Africa; 494 (38%) were women; 433 (33%) were men who have sex with men (MSM); and 694 (53%) were heterosexuals. Migrants had lower CD4 cell count at diagnosis than those born in France (296 vs 443, *P* < 0.01), likely due to delayed time to diagnosis. A total of 86 clusters were identified (clustering rate of 21%), including 33 of size ≥3 involving 170 participants (3–16 per cluster). MSM (OR 2.16, *P* < 0.01) and higher viral load (OR 1.21, *P* < 0.01) were risk factors of clustering. Individuals born abroad were clearly not detected in clusters, contrary to those born in France (OR 0.03, *P* < 0.01). Among large clusters, persistent virological control was achieved in a median of 75% of participants vs 83% outside clusters (*P* < 0.01). Molecular epidemiology showed that MSM were part of local transmission networks but not heterosexual migrants, suggesting distinct epidemic features and needs.

**IMPORTANCE:**

To end the HIV epidemic, it will also be necessary to halt the spread of the virus beyond large metropolitan areas, where resources are more limited and epidemiological trends are more difficult to grasp. In this study, we showed how molecular epidemiology could be used at the regional level to inform HIV surveillance and support decision-making for an appropriate local response. This could be especially helpful when national databases are lacking. We investigated the role and characteristics of transmission clusters in the HIV dynamics of the Centre-Loire Valley region, a low-density demographic and medical region near Paris, with a focus on migrants. We found that men who have sex with men (MSM) were part of local transmission clusters but not heterosexual migrants, suggesting distinct HIV epidemics and needs. Epidemic control would require further implementation of pre-exposure prophylaxis and treatment as prevention for MSM to slow the dynamics of HIV clusters locally, and generalized access to HIV screening for migrants.

## INTRODUCTION

Metropolitan areas play a critical role in HIV epidemics and therefore have been the target of much of the efforts in all areas of the HIV response. The Fast-Track Cities network, a global partnership supported by the Joint United Nations Program on HIV/AIDS, involves more than 350 cities from every region of the world committed to the Paris Declaration to End the AIDS Epidemic by 2030. Prevention and testing are of utmost importance to limit new contaminations. However, smaller urban areas as well as rural areas will also need to be taken care of to achieve this goal. While HIV testing, pre-exposure prophylaxis (PrEP), and treatment as prevention (TaSP) are universal tools, HIV response and efforts may need to be tailored to key population and specific aspects, based on knowledge of HIV dynamics within these territories.

Centre-Loire Valley (CLV) region in France is beyond the global goal for detection of the undiagnosed population, with one of the lowest rates of HIV testing (60/1,000 inhabitants) in France but among the highest rates for positive tests (1.4/1,000 HIV screening) after Paris region in the country, despite having one of the lowest populations (2.6 million inhabitants) and density (65.7 inhabitant/km^2^) in France (1). CLV also presents the lowest medical density with 350 physicians per 100,000 inhabitants. The region and its six departments (administrative subdivisions) may be linked to hotspots of HIV transmission fueled by the hidden epidemic. The introduction of PrEP in France in 2016 might have had variable impact, depending on the populations targeted, creating a gap in prevention access. Between 2015 and 2019, 40% of newly diagnosed people living with HIV (PLHIV) in CLV were men who have sex with men (MSM), and 43% were heterosexuals born in a different country (*N* = 487 new diagnoses), with local variations. Moreover, the PARCOURS survey suggests that HIV infection among migrants commonly occurs after their arrival in France and is linked to social hardship (2, 3), with poor access to prevention.

Molecular cluster identification has been proven to be an effective tool to identify key populations for tailored interventions (4, 5). Previous national phylogenetic analysis on individuals diagnosed at the acute stage has shown evidence that Paris was a central hub for HIV dissemination, especially subtype B and Circulating Recombinant Form (CRF) CRF02_AG, but those networks mainly implied that data on MSM and transmission networks in the migrant population are lacking (6, 7). Former studies have been able to identify clusters among migrants at the regional level (8–10). A better understanding of local epidemics can be accessed in region-wide studies, with locally growing cluster identification (11).

To identify and characterize HIV transmission networks in the CLV region, we performed phylogenetic analysis on all HIV *pol* sequences collected within the region over the last decade (2010–2020). We defined local risk factors of clustering and unraveled drivers of transmission dispersal to provide potential key public health interventions toward a better epidemic control.

## MATERIALS AND METHODS

### Study population and data collection

**Fig 1:**
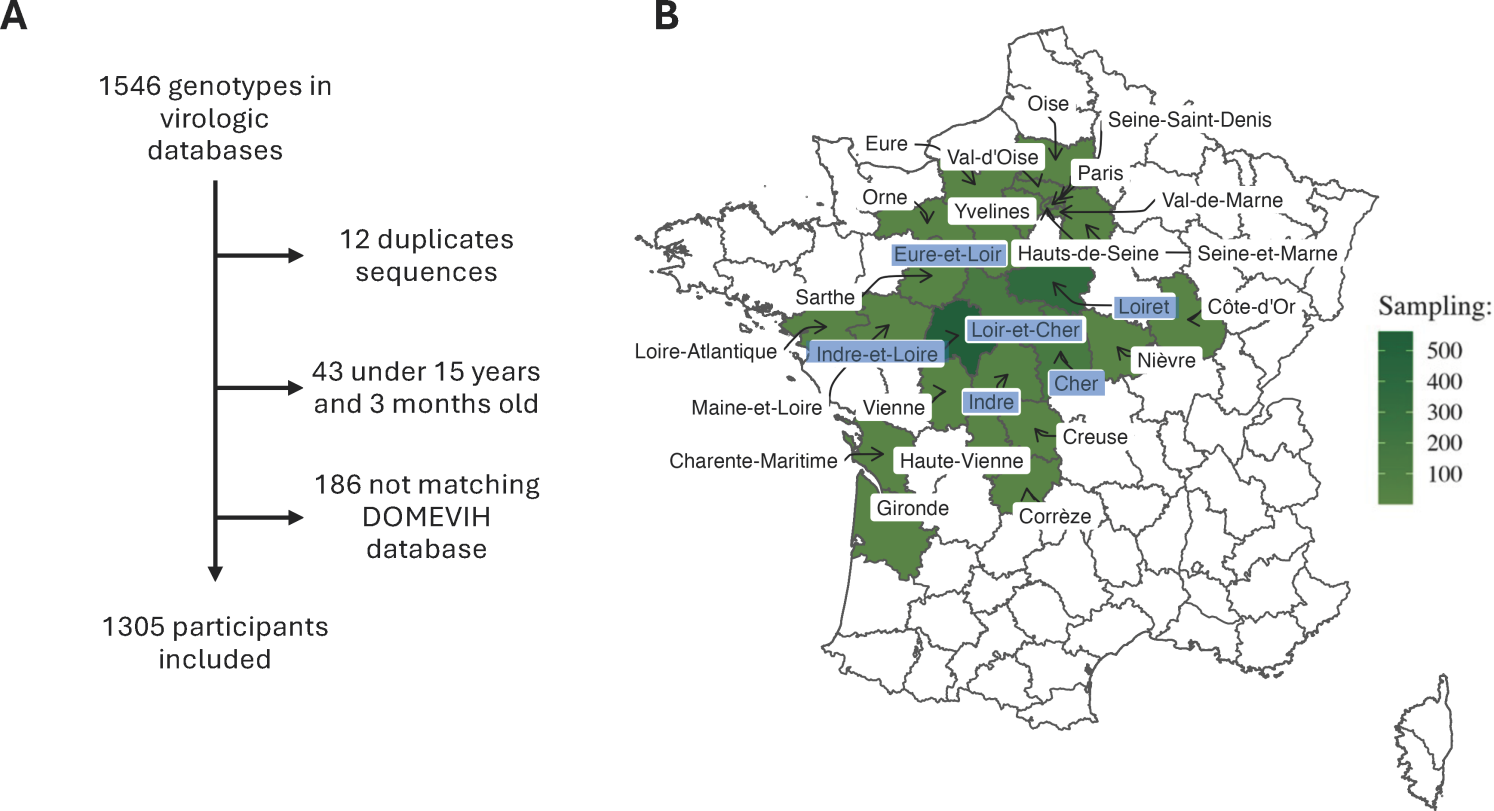
Flowchart (**A**) and sampling map overview (**B**). Each department (administrative division) is colored according to the number of individual sequences sampled in the data set, based on participants’ place of residence. The six departments of the Centre-Loire valley region are highlighted in blue.

We conducted an observational, retrospective, multicentric study using the DOMEVIH database, which includes clinical, laboratory, and epidemiological data from all patients followed in seven general hospitals of the CLV region (Fig. 1). All individuals aged 15 years and 3 months old or above, HIV infected, who agreed to DOMEVIH database inclusion, and had a drug-resistance genotyping between the years 2010a and 2020 were included in this study. We collected the clinical data available during this period (detailed in the Supplemental material). Persistent virological control after antiretroviral treatment (ART) initiation was estimated using the ratio of the number of undetectable viral loads to the number of viral load measurements after the first undetectable viral load. Sufficient virological control was defined as a ratio ≥90%, taking into account viral blips.

This research follows the recommended framework for phylogenetic analysis (12, 13). Clinical data were extracted by research assistants at each hospital in the CLV region and sent to a data manager at the Tours University Hospital. The data manager was responsible for matching clinical data and sequences and assigning an anonymization code. The anonymized database containing the sequences and metadata, but no identifying information (date of birth, home address, or identification number), was sent to the researchers responsible for the study to carry out the analyses. Access to the data was restricted to the researchers responsible for the research (K.S., A.C., N.C., and M.-F.T.). The clinical data of participants involved in clusters were not disclosed individually in order to minimize the risk of reidentification and stigmatization of participants when communicating molecular clusters.

### *Pol* gene sequencing

Drug resistance genotyping was realized in the two specialized virology laboratories of the CLV region (903 from Tours and 402 from Orléans) by sequencing the *pol* gene encoding HIV-1 reverse transcriptase and protease from patients’ plasma samples, using Sanger as previously described (14). Only one sequence per individual, the earliest available, was retained for the analysis. Of the 1,305 total sequences, 73 were short (ranging between 5 and 280 missing nucleotides); none showed high ambiguity; and 16 contained stop codons. None were excluded. HIV drug resistance for protease inhibitors (PIs), nucleoside reverse transcriptase inhibitors (NRTIs), and non-nucleoside reverse transcriptase inhibitors (NNRTIs) was assessed using the Stanford University HIV-1 genotypic resistance interpretation algorithm (https://hivdb.stanford.edu). HIV-1 subtypes were determined by uploading sequences individually into the REGA HIV-1 Automated Subtyping Tool version 3.47 and HIV Blast (https://www.hiv.lanl.gov/) (15). Recombinant sequences were detected using the bootscanning approach implemented in REGA, which scans the sequence in sliding windows to identify subtype breakpoints along the genome. This allowed classification of both pure subtypes and mosaic recombinant forms. In case of disagreement, a clade presenting >95% identity was retained, and complex recombinants were reported as unclassified, acknowledging that partial pol is imperfect for CRF identification. The sequence data have been uploaded on Dryad at https://doi.org/10.5061/dryad.m37pvmdgr. Metadata include sample identifier, date of sample collection, sequencing technology, consensus length, HXB2 coordinates, percent ambiguous bases, presence of frameshift, and premature stop codons.

### Transmission network reconstruction

We employed HIV Transmission Cluster Engine (HIV-TRACE, https://veg.github.io/hivtrace-wasm/) to infer transmission network clusters using *pol* gene sequencing (16). HIV-TRACE algorithm performs sequence alignment using bealign (Python 3, part of the BioExt library, https://github.com/veg/BioExt), with HXB2 pol as the reference and BLOSUM62 as the scoring matrix. All pairwise distances were calculated, and a putative linkage between each pair of two sequences was considered whenever their sequences were ≤1.5% distant (0.015 substitutions/site, TN93 substitution model), similarly to previous studies (6, 10, 17). The entire data set was processed in a single analysis, including all subtypes, and sensitivity analyses across genetic distance thresholds confirmed that 1.5% captures the largest number of distinct clusters without merging unrelated sequences (Fig. S1). Clusters comprising only two linked nodes were identified as dyads. Singletons were defined as individuals without any identified connection for the given genetic distance.

### Statistical analysis

Variations between the two groups were compared using Mann-Whitney’s unpaired test for quantitative variables. Contingency table analysis was performed using Fisher’s test for two proportion comparison and chi-square test for comparison of multiple proportions. For adjusted analysis, logistic regression was used to identify risk factors for being part of a cluster of *N* ≥ 3 (18).

A directed acyclic graph (DAG) representing the hypothesized causal relationships among the factors under investigation, as well as the unobserved factors, was initially constructed (see Fig. S2). The hypotheses associated with this DAG were assessed by examining the underlying conditional independences, considering up to two conditioning variables. The missing data in the database were subsequently imputed using a multiple imputation algorithm, employing a random forest method. The results of the analyses performed on complete cases are presented in the Supplemental material (see Fig. S3).

For each factor examined, a logistic regression model was applied to each of the 100 imputed data sets. Adjustments were made by considering the confounding factors delineated in the DAG. Additionally, a random intercept was estimated for each department of residence. The results from the 100 models were aggregated using Rubin’s rule to derive the final estimates. For all calculations, statistical significance was defined as a *P* value of <0.05. Statistical analyses were performed using both R software version 4.3 (mice version 3.18 package for multiple imputation and glmmTMB version 1.1.13 for regression) and GraphPad Prism version 9.1.1. A summary of model diagnostics is reported in the Supplemental material (see Table S1).

## RESULTS

### Population characteristics

Of the 1,546 available HIV genotypes, 1,305 participants matching the DOMEVIH database and age limit were then included in the phylogenetic analysis (Fig. 1). Available sequences mainly came from four departments of the CLV region: Indre-et-Loire (43%), Loiret (27%), Loir-et-Cher (11%), and Cher (11%) (Fig. 1). Table 1 displays the characteristics of the participants, including 38% of women, 33% of MSM, and 44% of people born in a foreign country, mainly sub-Saharan Africa (36%). Migrants had a lower CD4 T-cell count (296 vs 443 CD4/mm^3^ if born in France, *P* < 0,01) and were predominantly women (62%). Drug resistance mutations (DRMs) were present in 348 sequences (27%), including 46 (4%) with resistance to PI, 187 (14%) to NRTI, and 267 (20%) to NNRTI. The predominant subtype was subtype B (47%), followed by CRF02_AG (22%), and various other recombinant forms were detected, including unique recombinants (Table 2).

**TABLE 1:** Characteristics of clustered and non-clustered participants^*a*^

	Total	No cluster/dyads	Clusters (*n* ≥ 3)	*P* value
*N*	1,305	1,135	170	
Sex (F/M/T)	494 (38)/810 (62)/1	479 (42)/655 (58)/1	15 (9)/155 (91)	<0.01
Age at diagnosis				0.06
16–25	269 (21)	240 (21)	29 (17)	
26–35	472 (36)	420 (37)	52 (31)	
36–49	391 (30)	326 (29)	65 (38)	
≥50	173 (13)	149 (13)	24 (14)	
Department of residency				<0.01
Indre-et-Loire	560 (43)	483 (43)	77 (45)	
Loiret	358 (27)	329 (29)	29 (17)	
Loir-et-Cher	142 (11)	111 (10)	31 (18)	
Cher	148 (11)	124 (11)	24 (14)	
Others	97 (7)	88 (7)	9 (5)	
Contamination				<0.01
MSM	433 (33)	307 (27)	126 (74)	
Heterosexual	694 (53)	661 (58)	33 (19)	
PWID	67 (5)	63 (6)	4 (2)	
Others	35 (3)	33 (3)	2 (1)	
Unknown	76 (6)	71 (6)	5 (3)	
Birth country				<0.01
Metropolitan France	715 (55)	562 (50)	153 (90)	
Migrants	579 (44)	565 (50)	14 (8)	
French overseas	20 (2)	17 (1)	3 (2)	
Europe	31 (2)	27 (2)	4 (2)	
Sub-Saharan Africa	468 (36)	466 (41)	2 (1)	
North Africa	27 (2)	23 (2)	4 (2)	
Others	33 (3)	32 (3)	1 (1)	
Unknown	11 (1)	8 (1)	3 (2)	
Acute infection	97 (7)	67 (6)	30 (18)	<0.01
CDC category				0.02
A	1,038 (80)	891 (79)	147 (86)	
B	35 (3)	29 (3)	6 (4)	
C	232 (18)	215 (19)	17 (10)	
CD4 (cell count/mm^3^), median (±IQR)	371 (192–576)	365 (185–569)	417 (247–611)	0.06
Viral load (copies/mL), median (±IQR)	15,000 (141–110,000)	8,920 (75–96,600)	61,250 (8,883–195,250)	<0.01
Virological control				<0.01
Controlled	1,094 (84)	939 (83)	155 (91)	
Never undetectable	39 (3)	32 (3)	7 (4)	
Insufficient control	124 (10)	120 (11)	4 (2)	
Unknown	48 (4)	44 (4)	4 (2)	
Clade				<0.01
B	611 (47)	483 (43)	128 (76)	
02_AG	285 (22)	259 (23)	26 (15)	
06_cpx	21 (2)	11 (1)	10 (6)	
ART resistance	348 (27)	322 (28)	26 (15)	<0.01
Coinfection				
HBV	65 (5)	62 (5)	3 (2)	0.04
HCV	124 (10)	117 (10)	7 (4)	<0.01
STI	139 (11)	94 (8)	45 (26)	<0.01

^*a*^ Data are *N* (%), except if stated otherwise. CD4 and viral load are first available results in the DOMEVIH database. Virological control was defined as controlled if undetectable at the last visit or ≥90% of undetectable viral load since first undetectability; no follow-up was defined as strictly less than three viral load testing in the DOMEVIH database. ART resistance was defined as at least one class (PI, NRTI, or NNRTI) resistance following the Stanford algorithm. *P* values for univariate two-sided analysis between “no cluster/dyad” and “cluster” groups were calculated using Fisher’s exact test for two proportion comparisons, chi-square test for more than two proportion comparisons, or unpaired Mann-Whitney test for variations between two groups. MSM, men who have sex with men; PWID, people who inject drugs; STI, sexually transmitted infection.

**TABLE 2:** Distribution of subtypes and recombinant forms^*a*^

Subtype	Number of sequences	Number of clusters	Cluster size (number of clusters), including dyads
B	611	56	16 (*n* = 1), 10 (*n* = 2), 8 (*n* = 3), 6 (*n* = 2), 5 (*n* = 3), 4 (*n* = 2), 3 (*n* = 11), dyads (*n* = 32)
02_AG	285	15	7 (*n* = 1), 6 (*n* = 1), 4 (*n* = 1), 3 (*n* = 3), dyads (*n* = 9)
U	60	3	3 (*n* = 1), dyads (*n* = 2)
G	53	1	Dyad (*n* = 1)
C	44	2	Dyads (*n* = 2)
A1	38	1	Dyad (*n* = 1)
11_cpx	32		
A	24	1	Dyad (*n* = 1)
D	22		
01_AE	21		
06_cpx	21	2	10 (*n* = 1), dyad (*n* = 1)
A6	12		
H	12	1	Dyad (*n* = 1)
F1	10		
A3	8	1	Dyad (*n* = 1)
45_cpx	7	1	Dyad (*n* = 1)
F2	7	1	3 (*n* = 1)
25_cpx	6		
09_cpx	5		
13_cpx	4		
22_01A1	4		
37_cpx	4		
18_cpx	3		
27_cpx	3	1	Dyad (*n* = 1)
03_A6B	2		
07_BC	1		
14_BG	1		
26_A5U	1		
42_BF1	1		
49_cpx	1		
92_C2U	1		
94_cpx	1		

^*a*^ Final HIV-1 subtype classification based on combination of subtyping and recombination detection using REGA HIV-1 Automated Subtyping Tool version 3.47. Complex recombinant forms that did not match the current classification of existing CRF on the Los Alamos HIV database were reported as unclassified (U).

### Transmission networks and risk factors in clustered individuals

**Fig 2:**
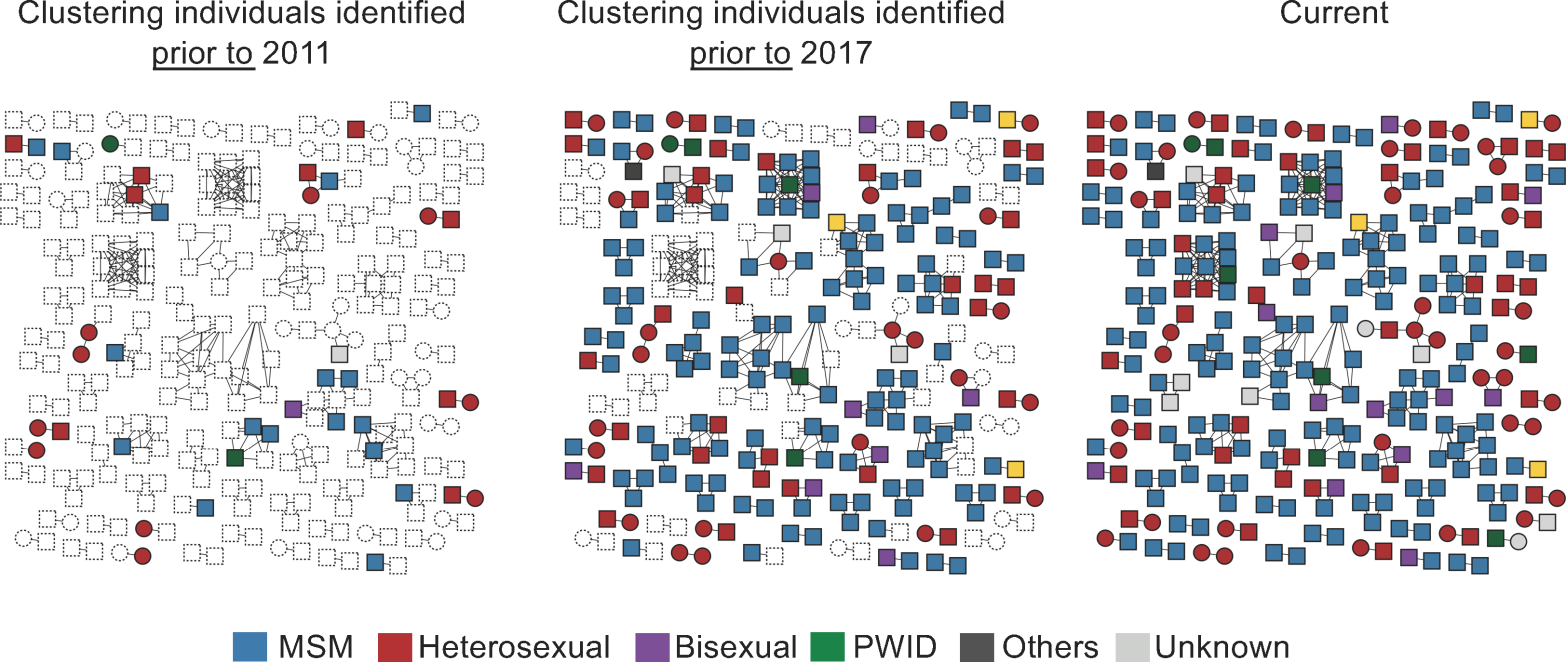
HIV transmission networks in the Centre-Loire valley region between 2010 and 2020. *N* = 86 clusters, including 53 dyads (*N* = 2) and 35 clusters of *N* ≥ 3. Each person is represented individually, with squares representing men and circles representing women. Lines connecting squares and circles represent pairwise distance between two individuals belonging to the same transmission cluster. A genetic distance threshold of 1.5% was applied to define clusters. Squares and circles are colored according to the known mode of HIV transmission (see legend in the figure) and the date of diagnosis (prior to 2011 on the left panel, prior to 2017 on the central panel, all cases on the right panel). New cases identified after 2017 are posterior to PrEP introduction in France. MSM, men who have sex with men; PWID, people who inject drugs.

We inferred a total of 86 clusters using a pairwise distance matrix with a genetic distance of less than 1.5% (Fig. 2), comprising 20.7% of the population. In total, 1,029 individuals were singletons (79%); 106 were clustered in dyads (8%); and 170 were in clusters of *N* ≥ 3 (13%, *n* = 33 clusters). Cluster size ranged between 16 (*n* = 1 cluster), 10–7 (*n* = 7 clusters), 6–4 (*n* = 9 clusters), and 3 individuals (*n* = 16 clusters). The repartition by subtype is detailed in Table 2. Multiple clusters connected individuals from different transmission risk groups. Several large transmission clusters have emerged or developed in the years since the implementation of PrEP in France in 2016.

DRM removal did not impact the network inferences. ART resistance was less frequent in clusters (15% vs 28%, *P* < 0.01), but shared resistance within a cluster was observed in six clusters (2 of *n* = 6, 2 of *n* = 4, 2 of *n* = 3). Individuals in clusters were more frequently diagnosed at acute infection (18% vs 6%, *P* < 0.01) and CDC stage A (86% vs 79%, *P* = 0.02) (Table 1).

**Fig 3:**
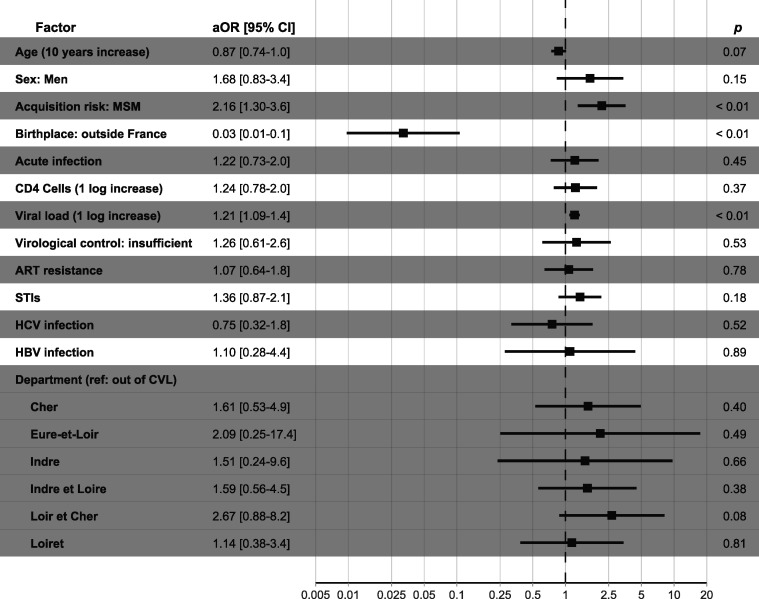
Multivariate logistic regression analysis of sociodemographic, clinical, and STI factors associated with HIV-1 transmission clusters. Risk factors of being part of a cluster of size *N* ≥ 3 were calculated in a multivariate analysis using logistic regressions on 100 imputed data sets. Insufficient virological control was defined as a ratio of undetectable viral loads to the total number of viral load measurements <0.9. MSM, men who have sex with men; STI, sexually transmitted infection (positive testing for syphilis, *Neisseria gonorrhoeae*, *Chlamydia trachomatis*, or reported diagnosis of any STI in medical records at any point of follow-up).

We performed a multivariate analysis to determine the risk factors associated with being part of a transmission cluster of size ≥ 3 (Fig. 3). MSM (OR 2.16, *P* < 0.01) and a higher first viral load (OR 1.21 per log_10_ copies of viral load, *P* < 0.01) were factors significantly associated with increased risk of clustering. On the other hand, participants born abroad were less at risk of clustering (OR 0.03, *P* < 0.01), and migrants were rarely detected in transmission clusters.

### Large cluster investigation

**Fig 4:**
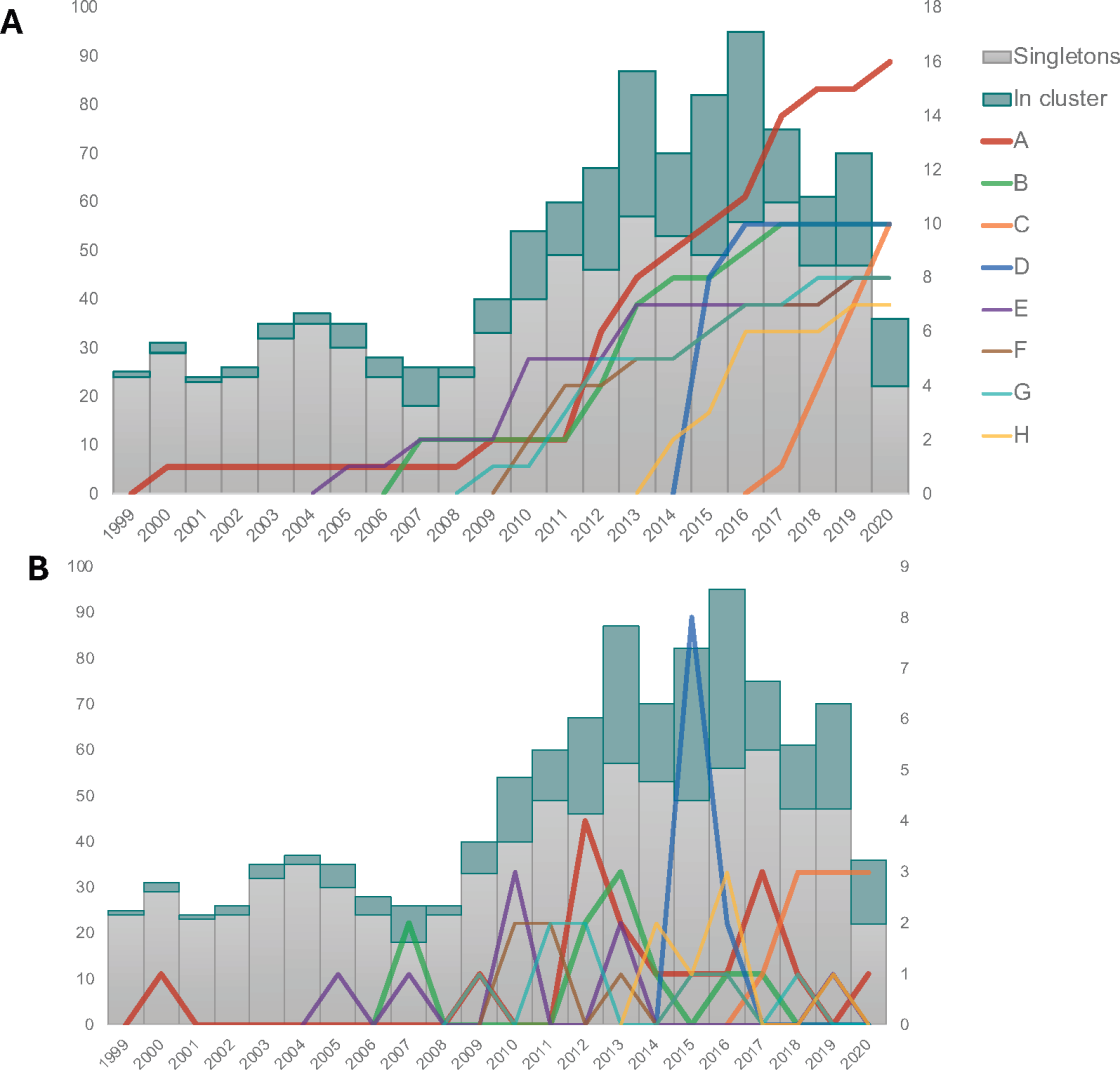
Longitudinal follow-up of clusters identified in CLV region’s sequence database between 2010 and 2020 with a focus on eight large clusters (*N* ≥ 7). (**A**) Cumulated growth and (**B**) growth per year of diagnosis. The size of the large clusters (A–H, colored according to the legend) is plotted on the right *Y*-axis. Each bar represents the total number of participants of the study by year of diagnosis (left *Y*-axis), with the proportion of participants in clusters (any size of cluster) in green and the proportion of participants not in clusters in gray.

Eight large clusters of *n* ≥ 7 individuals have been inferred, with individuals diagnosed between 2000 and 2020 (Fig. 4; Table 3). Our data set was more effective to infer clusters for individuals diagnosed after 2013. The largest cluster (A, *n* = 16) had the first individual diagnosed in 2000, peaked in 2012, and continued to grow through 2020, mainly in Indre-et-Loire (94%). Cluster C (clade 06_cpx) had ongoing new diagnoses since its first case in 2017 and spread in at least half of the CLV region. Cluster D new cases emerged in 2015 in Loiret (100%), but no new case has been diagnosed since 2016. We investigated the role of unsuppressed viremia despite ART in the spread of transmission clusters. Persistent virological control was less often observed in participants involved in those large clusters compared to non-clustered individuals (75% versus 83%, *P* < 0.01). Cluster D was the only large cluster with 100% of people with sufficient virological control and was short-lived in our study period.

**TABLE 3:** Characteristics of large clusters (*N* ≥ 7)^*a*^

Cluster	*N*	Clade	MSM (%) Migrants (%) PWID (%)	Residency (%)	≥90% undetectability ratio (%)	Coinfection (%)
A	16	B	13 (81) 1 (6) 1 (6)	IL (94) LC (6)	12 (75)	STI: 5 (31) HBV: 1 (6)
B	10	B	9 (90) 0 0	LC (50) IL (20) S (20) C (10)	9 (90)	STI: 2 (20)
C	10	06_cpx	6 (60) 2 (20) 1 (10)	LC (67) IL (22) L (11)	7 (70)	STI: 3 (30) HBV: 1 (10)
D	10	B	8 (80) 2 (20) 1 (10)	L (100)	10 (100)	STI: 4 (40)
E	8	B	8 (100) 0 0	C (100)	6 (75)	STI: 2 (25) HCV: 1 (13)
F	8	B	6 (75) 1 (13) 0	IL (50) L (38) LC (12)	6 (75)	STI: 2 (25)
G	8	B	8 (100) 0 0	IL (75) EL (25)	5 (63)	STI: 5 (63)
H	7	02_AG	6 (86) 1 (14) 0	IL (57) C (29) G (14)	6 (86)	STI: 2 (29)

^*a*^ All large cluster members were male. C, Cher; EL, Eure-et-Loir; G, Gironde; IL, Indre-et-Loire; L, Loiret; LC, Loir-et-Cher; MSM, men who have sex with men; PWID, people who inject drugs; S, Sarthe; STI, sexually transmitted infection.

## DISCUSSION

We realized a regional-scale study in CLV, a sparsely populated region at the gateway to Paris, to improve insight on HIV epidemic dynamics and drivers of transmission to organize targeted interventions. CLV region clusters were largely composed of MSM, born in France, more frequently diagnosed during acute infection, with higher viral loads and subtype B infection, similar to other Western European countries (18, 19). Migrants were remarkably not detected in the regional transmission networks based on genetic distance analysis. A history of STI was common and, as previously suggested, could be a marker of growing transmission clusters (20). We also show that 14 clusters emerged or expanded in CLV despite PrEP implementation in 2016, underlining the need to improve its access for key populations across the region.

One of our primary focuses was to better understand the determinants of HIV epidemics among migrants who represent about 40% of PLHIV in the region. Despite this high proportion among new diagnostics, our study shows that they were clearly not detected in transmission clusters based on viral genetic distance. This does not mean that local transmission is strictly absent, but rather that it is less frequent or that it goes unnoticed with this method, which has epidemiological implications. Singletons may not have been connected to any clusters for several reasons that could coexist. First, HIV transmission chains include undiagnosed individuals, participating in the hidden epidemic. Second, cluster inference was based on a short genetic distance (1.5%) ruling out a link between two individuals whose transmission occurred longer time ago, with greater individual viral evolution, consistent with the higher proportion of late-presentation diagnosis at CDC stage C. Third, other clades such as CRF02_AG may have a different evolutionary rate than subtype B, which cannot be taken into account in this analysis (21). Of note, the epidemiological profile of people born outside metropolitan France differed in many ways: they were mainly from sub-Saharan Africa, had a higher proportion of women, had more frequent heterosexual transmission, had lower CD4 at diagnosis, and had a higher prevalence of CRF02_AG viruses (Table S2). Our data cannot address if contamination happened outside the CLV region (in their birth country or during their migration process). However, a phylogeographic analysis identified clades with greater genetic distance but specific to the CLV region, which enabled us to infer transmission events between individuals from different origins and in multiple directions (Fig. S4; Table S1). This may be a small but significant signal that this population is involved in the hidden epidemic within our region, reinforcing the suspicion from previous surveys that up to half of them have contracted HIV upon arrival (2).

Due to differences in the epidemic profile of MSM and migrants in the CLV region, public health responses may need to differ, depending on the target population. Migrants, who present less frequently with STIs associated with at-risk sexual behavior, but more frequently at late-stage diagnosis, are probably an important component of the undiagnosed population. They should benefit first from improved screening programs to close the gap in the HIV care continuum. MSM, on the contrary, seem to be well reached by the HIV screening strategy but are at increased risk of infection through transmission clusters with at-risk sexual behavior. Accelerating the implementation of PrEP would likely have a much greater impact in reducing this risk and blocking transmission chains. The same actions are promoted by the *Paris Sans SIDA* initiative and should be extended to neighboring territories. Outside major metropolitan areas where fast-track HIV policies are implemented, low medical density could lead to additional challenges, such as linkage to care and long-term adherence to treatment. Indeed, we observed that clusters that continued to expand over the past decade also included individuals with insufficient virological control, who may have contributed to cluster expansion. This emphasizes the importance of TASP. Remote consultation may have an important impact in such context (22). Importantly for regional surveillance, we detected a large cluster of a less frequent clade, CRF06_cpx, with only 70% of estimated virological control since first undetectability. It was still expanding in 2024 (unpublished surveillance data) and is currently the subject of a national investigation. This illustrates the usefulness of cluster analyses on a regional scale, despite their lower power compared to nationwide studies.

Our data set, although limited to an administrative region, provided 1,305 sequences over a decade, enabling robust phylogenetic analysis. The incidence was estimated by the French National Public Health Agency between 2015 and 2019 at values ranging from 98 to 119 (4.0–4.8 per 100,000), and this study included 66%–70% of the new infections during this period. However, some centers were undersampled as they could outsource this test outside the region. Data from different centers also differed over the time period, underpowering potential dynamics before 2013 and after 2018. Similarly to other studies using phylogenetic networks, the interpretation of our results is limited by data completeness, which underestimates the number and the size of the clusters. Different subtypes may present variable evolutionary rates, and a detailed analysis of all the recombinant forms detected in the cohort could help better describe the complex evolution of all the lineages, as illustrated by the study of Switzer et al. describing an outbreak among persons who inject drugs (23).

In conclusion, molecular epidemiology has the potential to guide tailored public health measure implementation, even locally with region-scaled analyses where national molecular surveillance is lacking. Real-time phylogenetic identification and notification of clusters may help limit the spread of HIV in the future (24), in conjunction with clinicians caring for PLHIV, as well as field actors to link with communities.

## SUPPLEMENTAL MATERIAL

Supplemental material spectrum.02542-25-s0001.docx Additional experimental details, Figures S1 to S4, and Tables S1 to S3.

ASM does not own the copyrights to Supplemental Material that may be linked to, or accessed through, an article. The authors have granted ASM a non-exclusive, world-wide license to publish the Supplemental Material files. Please contact the corresponding author directly for reuse.
