# Supplementary material for: HIV epidemic and needs beyond fast-track cities: a transmission network analysis to study the dynamics of HIV clusters in a French region near Paris
Source: Microbiol Spectr. 2026 Apr 22;14(6):e02542-25. doi: 10.1128/spectrum.02542-25 (PMC13227971; doi:10.1128/spectrum.02542-25)
Supplement: Supplemental material — Additional experimental details, Figures S1 to S4, and Tables S1 to S3. [file spectrum.02542-25-s0001.docx]

**Supplementary Material**

**Collected data**

age at diagnosis; sex; geographic department (administrative subdivision) of residency; birthplace; CDC HIV infection category at diagnosis; risk group; date of diagnosis (defined as date of first available date of seropositivity or self-declared); CD4 counts and HIV-1 viral loads; HBV or HCV co-infection; associated STI (positive testing for syphilis, *Neisseria gonorrhoeae*, *Chlamydia trachomatis*, or reported diagnosis of any STI in medical records) at any point of follow-up.

**Sensitivity analyses across genetic distance.**

Comprehensive sensitivity analyses across genetic distance (GD) cutoffs from 0.5% to 3.5%, using two ambiguity-resolution fractions (0.015 and 0.05) and with/without DRM stripping. These analyses were done as previously reported [1,2]and are illustrated in Figure S1.

• At the GD used for our main analysis (1.5%), the overall network structure is essentially unchanged by DRM removal: the number of nodes and edges changed by 0.5 % 354 vs 356 nodes; and 1.9 % change in edges (464 vs 473), respectively; the number of clusters changed from 84 (raw) to 85 (DRM-stripped), and the maximum cluster size did not change

• We compared HIV cluster membership at a 1.5% genetic distance cutoff between networks generated with and without DRM stripping. Using sequences common to both analyses, we computed the Adjusted Rand Index (ARI), Jaccard index, and KS test on cluster size distributions. The ARI was 0.978, indicating near-identical clustering between the two approaches. The Jaccard index was 0.958, confirming a high degree of sequence-level cluster overlap. The KS test comparing cluster size distributions returned a p-value of 1, indicating no significant difference. Overall, these results demonstrate that DRM stripping had minimal impact on cluster composition at this cutoff.

• Together, these results show that DRMs do not materially drive cluster detection in our dataset at the GD threshold used for the main analysis; the small number of edge/node changes observed are consistent with expected stochastic effects of site removal rather than systematic DRM-driven clustering.

We selected a genetic distance (GD) cutoff of 1.5% as a balance between detecting the maximum number of clusters while avoiding cluster coalescence, which would artificially inflate cluster sizes. Sensitivity analyses across GD thresholds confirmed that 1.5% captures the largest number of distinct clusters without merging unrelated sequences or reflecting older putative transmission events.

As distance thresholds increase, smaller clusters merge into larger, less informative clusters (see figure , red lines). At the extreme, all sequences would belong to a single cluster, which while technically correct, since all HIV-1 sequences are related through a series of transmissions, this finding is unlikely to be of interest in the context of molecular epidemiology.

**Identification of representative phylogenetic clades**

After sequence curation, transmission networks that best approximate the epidemic dynamics were identified according to Cuypers *et al*. [3]. CLV region HIV partial *pol* sequences were complemented with available location-annotated publicly available HIV *pol* sequences from other countries. We used BLAST to select the 50 closest genomes to each of our sequences in order to maintain the most relevant set of background sequences [4]. This selection accounted for a total of 6,728 sequences outside of CLV region, for a total of 8033 sequences in background dataset. These sequences were aligned to the HXB2 pol reference sequence (GenBank accession K03455). Next, phylogenetic trees were inferred using FastTree2 under the GTR+Γ substitution model [5]. From these trees, well-supported clades (i.e. Shimodaira Hasegawa (SH) local support of at least 0.9) including only sequences from CLV were identified [6].

**Phylogeographic inference**

Phylogeographic inference was performed using the asymmetric discrete phylogeographic model implemented in the BEAST 1.10.5 software package [7]. To promote estimation accuracy and precision of the migration rates and the nucleotide substitution rates, the migration and substitution model (GTR+Γ) were shared across the clades [8]. Estimates of the expected number of migration events between all pairs of locations (Markov jumps) were computed through stochastic mapping techniques [9]. Specifically, we looked at migration from outside the study region to the study region (viral introductions) and migration within the region (between French departments). Sampling uncertainty was allowed by assigning missing risk group information as an ambiguous risk that can take the value of the different risk groups. The starting state of a change in location or risk group is referred to as “from” and where the virus migrated is referred to as the “to”.

In the absence of clear temporal signal, time-calibrated inferences are unreliable, but evolutionary rate parameter can commonly be estimated by using independently derived evolutionary rate parameters [10]. For subtype B (47% of our cohort), the evolutionary rate for *pol* is estimated between ~0.001 and ~0.003 substitutions/site/year (s/s/y). For this reason, we specified a normal distribution as prior on the mean clock rate with mean 0.002 s/s/y and standard deviation such that the 95% CI ranges from 0.001 s/s/y to 0.003 s/s/y. We used a relaxed clock model in clades with ≥10 taxa and a strict clock model was assumed for smaller clades. A constant size coalescent tree prior was specified for all clades.

To interrogate HIV dispersal within the region, we analyzed all clades of size ≥ 3 sequences (n=56). MCMC chains were run sufficiently long to ensure adequate mixing. Maximum clade credibility (MCC) trees were obtained with TreeAnnotator 1.10 and convergence and mixing properties were inspected using Tracer 1.7. Using the same approach, we developed additional discrete phylogeographic models using the country of origin as main trait and risk group to interrogate the spread across risks between individuals from different origin. To interrogate HIV dispersal across individuals of different origins, we analyzed all clades of size ≥ 3 sequences (n=38).

**Supplementary Figure S1. Sensitivity analyses across genetic distance (GD) cutoffs from 0.5% to 3.5%, using two ambiguity-resolution fractions (0.015 and 0.05) and with/without DRM stripping.**


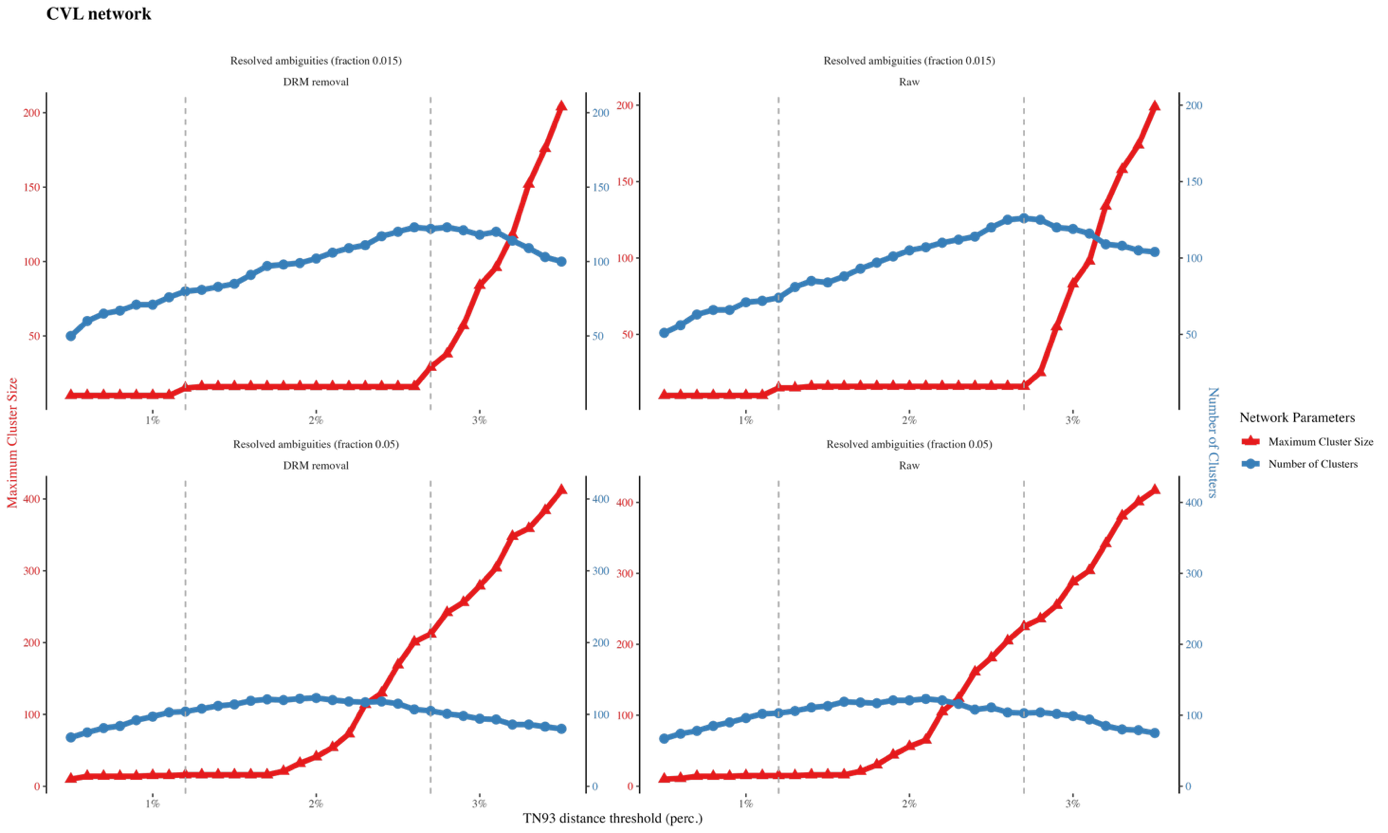


**Supplementary Figure S2: Directed acyclic graph**


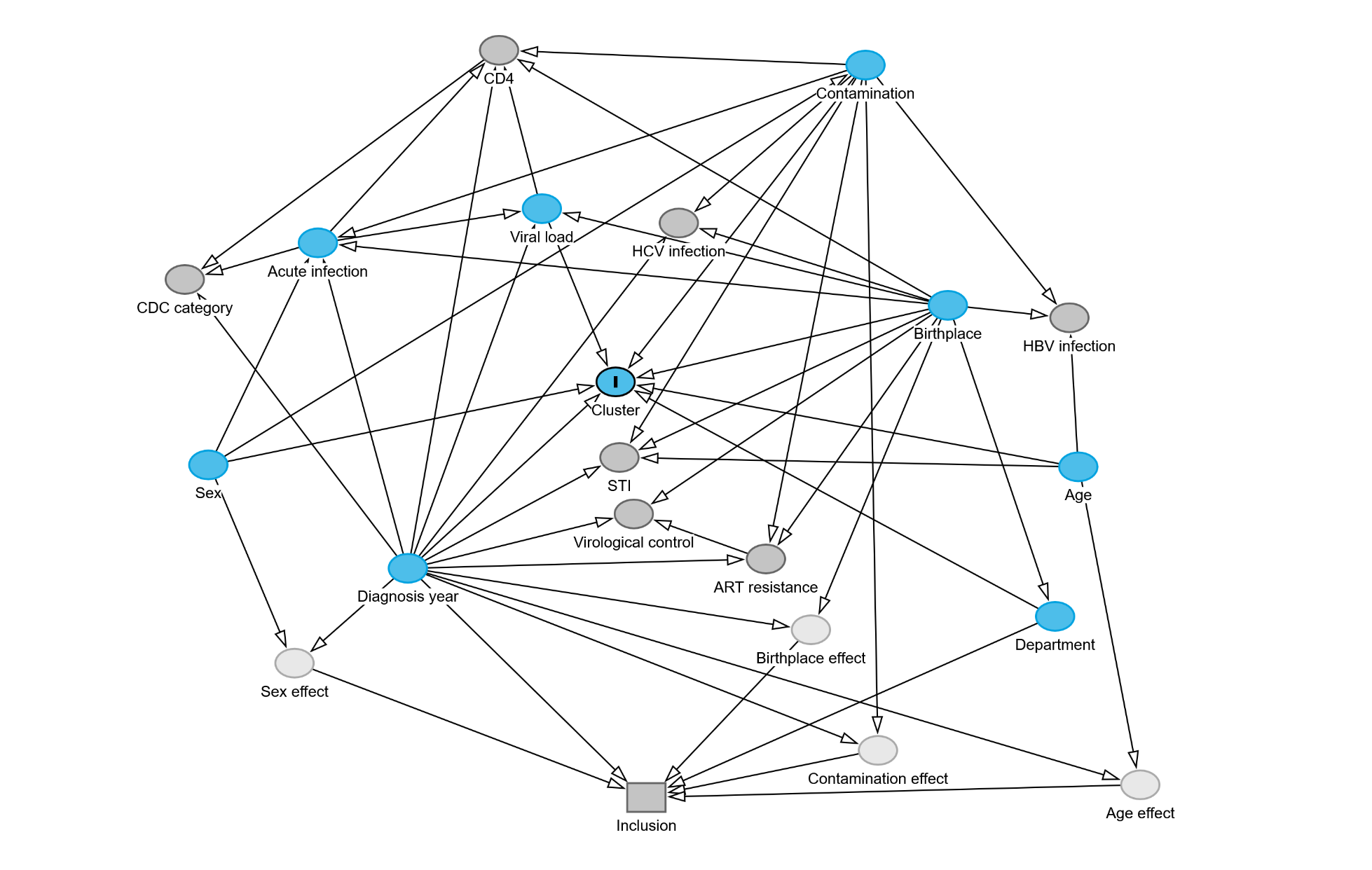


**Supplementary Figure S3: Comparisons of results obtained from imputed data and complete cases for the adjusted estimate of the association between individual characteristics and the risk of cluster membership**

**
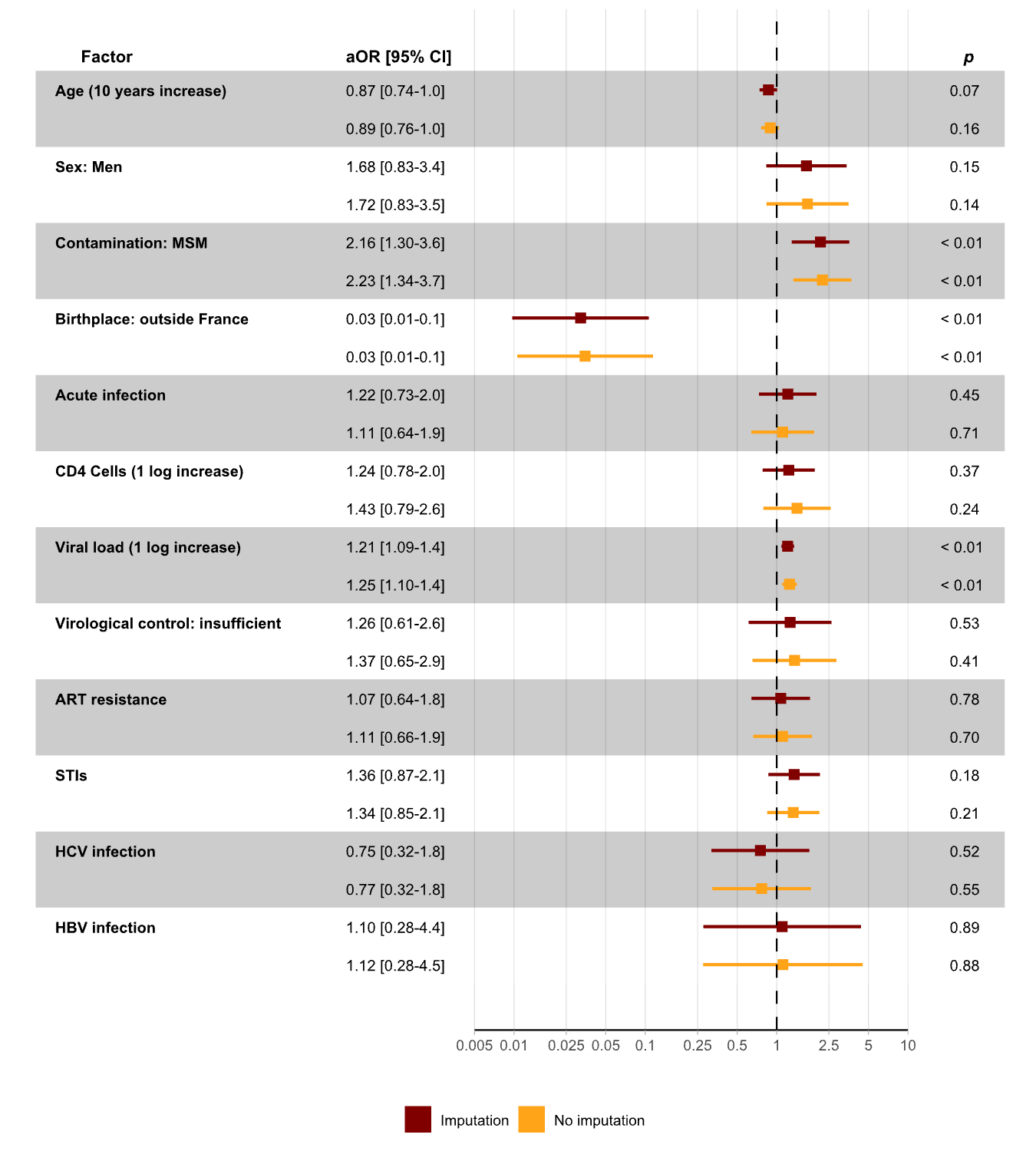
**

**Supplementary Figure S4. Dispersal between individuals from different origins.** Circle sizes are proportional to the number of supported events (BF_adj_ ≥ 3). HTS = Heterosexual.


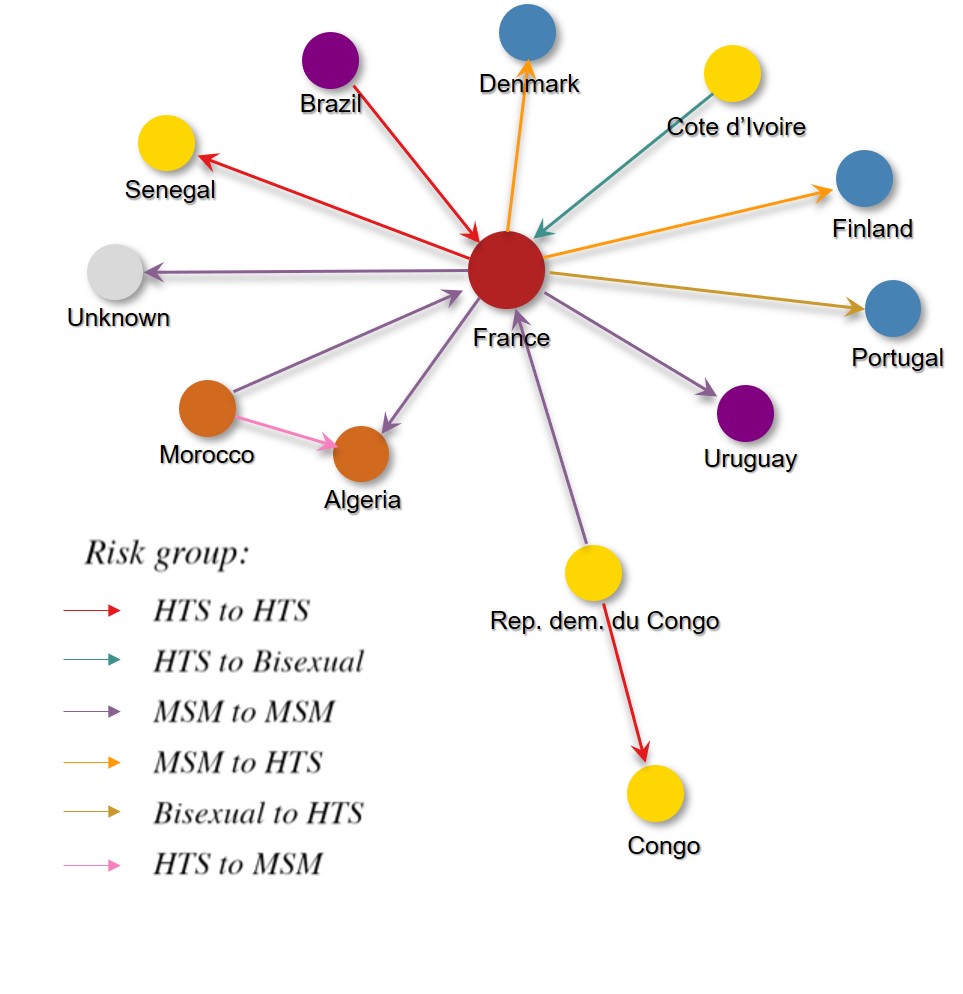


**Supplementary Table S1. Diagnostics summary of regression models.** For each model the table presents the risk factors that were investigated along with regression covariates. The Kolmogorov-Smirnov test assesses the deviation from the assumption of uniformity of simulated residuals, which should be flat regardless of the number of hierarchical levels involved in the model. The Shapiro test assesses the deviation from the assumption of normality of random effects. The Variance Inflation Factor (VIF) measures how much multicollinearity inflates the variance of regression coefficients. A VIF >= 5 indicates a multicolineraty issue. All test were performed on the model built on the first imputed dataset.

| Model | Risk factor investated | Covariates | Uniformity of simulated residuals (Kolmogorov-Smirnov test) | Normality of random effects (Shapiro test) | Colinearity |
| --- | --- | --- | --- | --- | --- |
| Model 1 | Age, Sex, Brithplace | Age, Sex, Birthplace, Contamination, Year, Department (random effect) | p = 0.94 | p = 0.19 | all VIF <= 1.23 |
| Model 2 | Acute infection | Contamination, Birthplace, Year, Department (random effect) | p = 0.97 | p = 0.212 | All VIF <= 1.22 |
| Model 3 | CD4 | Acute infection, Viral load, Birthplace, Contamination, Year, Department (random effect) | p = 0.81 | p = 0.24 | All VIF <= 0.94 |
| Model 4 | Viral load | Acute infection, Birthplace, Year, Department (random effect) | p = 0.80 | p = 0.6 | All VIF <= 0.99 |
| Model 5 | Virological control | Birthplace, Contamination, Year, Department (random effect) | p = 0.93 | p = 0.28 | All VIF <= 0.96 |
| Model 6 | ART resistance | Birthplace, Contamination, Year, Department (random effect) | p = 0.94 | p = 0.25 | All VIF <= 0.94 |
| Model 7 | STIs | Age, Birthplace, Contamination, Year, Department (random effect) | p = 0.95 | p = 0.16 | All VIF <= 0.94 |
| Model 8 | HCV infection | Birthplace, Contamination, Year, Department (random effect) | p = 0.93 | p = 0.26 | All VIF <= 0.97 |
| Model 9 | HBV infection | Birthplace, Contamination, Year, Department (random effect) | p = 0.91 | p = 0.22 | All VIF <= 0.98 |
| Model 10 | HBV infection | Sex, Age, Birthplace, Contamination, Year | p = 0.33 | - | All VIF <= 0.94 |

**Supplementary Table S2.** **Migrant population characteristics.** CD4 and viral load are first available results in DOMEVIH database. Virological control was defined as : controlled if undetectable at last visit or ≥ 90% of undetectable viral load since first undetectability ; no follow up was defined as strictly less than 3 viral load testing in DOMEVIH database. ART resistance was defined as at least one class (PI, NRTI or NNRTI) resistance following Stanford algorithm. *p*-values for univariate two-sided analysis between “No cluster / dyads” and “clusters” groups were calculated using Fisher’s exact test for 2 proportions comparisons, Chi² test for more than 2 proportions comparisons or unpaired Mann-Whitney test for variations between 2 groups.

|  | **Migrants** | **Born in**  **Metropolitan France** | ***p*-value** |
| --- | --- | --- | --- |
| **N** | 579 | 715 |  |
| **Sex (F/M/T)** | 357 (62) / 221 (38) / 1 | 131 (18) / 584 (82) | **< 0.01** |
| **Age at diagnosis** |  |  | **< 0.01** |
| 16-30 | 250 (43) | 265 (37) |  |
| 31-49 | 273 (47) | 334 (47) |  |
| ≥ 50 | 56 (10) | 116 (16) |  |
| **Department of residency** |  |  | **< 0.01** |
| Indre-et-Loire | 232 (40) | 328 (46) |  |
| Loiret | 217 (37) | 137 (19) |  |
| Loir-et-Cher | 50 (9) | 87 (12) |  |
| Cher | 42 (7) | 105 (15) |  |
| Indre | 12 (2) | 18 (3) |  |
| Eure-et-Loir | 7 (1) | 6 (1) |  |
| Sarthe | 4 (1) | 11 (2) |  |
| Others | 11 (2) | 23 (3) |  |
| **Contamination** |  |  | **< 0.01** |
| MSM | 39 (7) | 393 (55) |  |
| Heterosexual | 468 (81) | 219 (31) |  |
| PWID | 19 (3) | 48 (7) |  |
| Others | 9 (2) | 15 (2) |  |
| Unknown | 44 (8) | 40 (6) |  |
| **Acute infection** | 13 (2) | 84 (12) | **< 0.01** |
| **CDC category** |  |  | 0.30 |
| A | 454 (78) | 575 (80) |  |
| B | 13 (2) | 22 (3) |  |
| C | 112 (19) | 118 (17) |  |
| **CD4 (cell count/mm^3^)** | 296 (150-468) | 443 (234-636) | **< 0.01** |
| **Viral load (copies/ml) median (± IQR)** | 13,205 (225-101,500) | 15,000 (78-120,000) | 0.92 |
| **Virological control** |  |  | **< 0.01** |
| Controlled | 456 (79) | 630 (88) |  |
| Never undetectable | 25 (4) | 13 (2) |  |
| Insufficient control | 72 (12) | 51 (7) |  |
| No follow up | 26 (4) | 21 (3) |  |
| **Clade** |  |  | **< 0.01** |
| B | 63 (11) | 545 (76) |  |
| 02_AG | 199 (34) | 84 (12) |  |
| 06_cpx | 12 (2) | 8 (1) |  |
| **ART resistance** | 168 (29) | 179 (25) | 0.11 |
| **Coinfection** |  |  |  |
| HBV | 53 (9) | 12 (2) | **< 0.01** |
| HCV | 44 (8) | 79 (11) | **0.04** |
| STI | 14 (2) | 72 (10) | **< 0.01** |

**Supplementary Table S3. Dispersal between individuals from different origins.** BF = Bayes factor. BF_adj_ = Adjusted Bayes factor.

| **HIV transmission events** | | | | |
| --- | --- | --- | --- | --- |
| **Centre-Val de Loire region, France** | | | | |
| **Country of origin** | **Country of destination** | **Events** | **BF** | **BF_adj_** |
| Algeria | Guadeloupe | 0.3 (0.7%) | 5.2 | 3.7 |
|  | Portugal | 0.1 (0.2%) | 3.3 | 5.5 |
| Brazil | France | 1.4 (3.5%) | 26 | 5.9 |
| Côte d’Ivoire |  | 4.3 (10.6%) | 551.8 | 43.7 |
| France | Algeria | 4.9 (12.1%) | 19,696.6 | 23.0 |
|  | Brazil | 0.1 (0.2%) | 3.1 | 3.7 |
|  | Denmark | 0.2 (0.5%) | 4.6 | 6.9 |
|  | Finland | 0.7 (1.7%) | 26.0 | 4.1 |
|  | Unknown | 5.1 (1.2%) | 39,421.5 | 46.0 |
|  | Portugal | 0.5 (1.2%) | 14.6 | 91.3 |
|  | Senegal | 1.1 (2.7%) | 52.9 | 25.2 |
|  | Uruguay | 1 (2.5%) | 46.6 | 93.0 |
| Unknown | Denmark | 0.2 (0.5%) | 5.7 | 3.4 |
|  | Portugal | 0.2 (0.5%) | 6.3 | 6.0 |
| Morocco | Algeria | 1 (2.5%) | 29.1 | 37.2 |
|  | France | 5.9 (14.6%) | 702.2 | 56.7 |
| Rep. Dem. Du Congo | Congo | 5.3 (13.1%) | 569.4 | 4.2 |
|  | France | 7.8 (19.3%) | 519.6 | 3.1 |
| Roumania | Denmark | 0.1 (0.2%) | 3.6 | 4.1 |
|  | Portugal | 0.1 (0.2%) | 3.0 | 3.8 |
| Senegal | Mayotte | 0.1 (0.2%) | 4.1 | 3.0 |
| Uruguay | Portugal | 0.1 (0.2%) | 3.1 | 3.8 |

**REFERENCES**

[1] Wertheim JO, Pond SLK, Forgione LA, Mehta SR, Murrell B, Shah S, et al. Social and Genetic Networks of HIV-1 Transmission in New York City. PLOS Pathogens 2017;13:e1006000. https://doi.org/10.1371/journal.ppat.1006000.

[2] Chen Y, Lan G, Feng Y, Ruan Y, Shen Z, McNeil EB, et al. Inferring potential non-disclosed men who have sex with men among self-reported heterosexual men with HIV in Southwest China: A genetic network study. PLoS One 2023;18:e0283031. https://doi.org/10.1371/journal.pone.0283031.

[3] Cuypers L, Vrancken B, Fabeni L, Marascio N, Cento V, Di Maio VC, et al. Implications of hepatitis C virus subtype 1a migration patterns for virus genetic sequencing policies in Italy. BMC Evolutionary Biology 2017;17:70. https://doi.org/10.1186/s12862-017-0913-3.

[4] Dereeper A, Audic S, Claverie J-M, Blanc G. BLAST-EXPLORER helps you building datasets for phylogenetic analysis. BMC Evol Biol 2010;10:8. https://doi.org/10.1186/1471-2148-10-8.

[5] Price MN, Dehal PS, Arkin AP. FastTree 2 – Approximately Maximum-Likelihood Trees for Large Alignments. PLOS ONE 2010;5:e9490. https://doi.org/10.1371/journal.pone.0009490.

[6] Shimodaira H, Hasegawa M. CONSEL: for assessing the confidence of phylogenetic tree selection. Bioinformatics 2001;17:1246–7. https://doi.org/10.1093/bioinformatics/17.12.1246.

[7] Suchard MA, Lemey P, Baele G, Ayres DL, Drummond AJ, Rambaut A. Bayesian phylogenetic and phylodynamic data integration using BEAST 1.10. Virus Evolution 2018;4:vey016. https://doi.org/10.1093/ve/vey016.

[8] Drummond AJ, Ho SYW, Phillips MJ, Rambaut A. Relaxed Phylogenetics and Dating with Confidence. PLOS Biology 2006;4:e88. https://doi.org/10.1371/journal.pbio.0040088.

[9] Minin VN, Suchard MA. Counting labeled transitions in continuous-time Markov models of evolution. J Math Biol 2008;56:391–412. https://doi.org/10.1007/s00285-007-0120-8.

[10] Nasir A, Dimitrijevic M, Romero-Severson E, Leitner T. Large Evolutionary Rate Heterogeneity among and within HIV-1 Subtypes and CRFs. Viruses 2021;13:1689. https://doi.org/10.3390/v13091689.
